# RSV Infection in Neonatal Mice Induces Pulmonary Eosinophilia Responsible for Asthmatic Reaction

**DOI:** 10.3389/fimmu.2022.817113

**Published:** 2022-02-02

**Authors:** Dan Zhang, Jie Yang, Yuanhui Zhao, Jinjun Shan, Lingling Wang, Guang Yang, Susu He, Erguang Li

**Affiliations:** ^1^State Key Laboratory of Pharmaceutical Biotechnology, Medical School, Nanjing University, Nanjing, China; ^2^Jiangsu Key Laboratory of Molecular Medicine, Medical School, Nanjing University, Nanjing, China; ^3^Yancheng Medical Research Centre, Medical School, Nanjing University, Yancheng, China; ^4^Institute of Medical Virology, Drum Tower Hospital, Medical School, Nanjing University, Nanjing, China; ^5^Centre of Pediatric Diseases, College of Clinical Medicine, Nanjing University of Chinese Medicine, Nanjing, China; ^6^Nanjing Children’s Hospital, Nanjing Medical University, Nanjing, China

**Keywords:** RSV, asthma, adoptive transfer, eosinophilic inflammation, eosinophilia

## Abstract

Respiratory syncytial virus (RSV) is a leading cause of lower respiratory tract infections in infants and young children. Severe respiratory viral infection in early life is intimately associated with childhood recurrent wheezing and is a risk factor for asthma later in life. Although eosinophilic airway inflammation is an important trait in asthma of children, the roles of pulmonary eosinophils in the disease have been inadequately understood. Here, we show that RSV infection in neonatal mice causes eosinophilia after allergen stimulation. We showed that RSV infection in neonatal mice exacerbated allergic asthma to allergen stimulation that was accompanied with increased detection of eosinophils in the lungs. In addition, we also detected accumulation of ILC2, CD4^+^ T cells, and macrophages. Importantly, adoptive transfer of eosinophils from asthmatic mice with early-life RSV infection exacerbated pulmonary pathologies associated with allergic respiratory inflammation in naive mice in response to foreign antigen. The induction of asthmatic symptoms including AHR, tracheal wall thickening, and mucus production became more severe after further stimulation in those mice. The expression of antigen presentation-related molecules like CD80, CD86, and especially MHC II was markedly induced in eosinophils from OVA-stimulated asthmatic mice. The accumulation of CD4^+^ T cells in the lungs was also significantly increased as a result of adoptive transfer of eosinophils. Importantly, the deterioration of lung pathology caused by adoptive transfer could be effectively attenuated by treatment with indomethacin, a nonsteroidal anti-inflammatory drug. Our findings highlight the significance of eosinophil-mediated proinflammatory response in allergic disease associated with early-life infection of the respiratory tract.

## Introduction

Asthma is characterized by increased airway hyperresponsiveness (AHR), inflammatory infiltrates, and airway remodeling, which can be any combination of symptoms like cough, wheeze, and shortness of breath and chest tightness. It was estimated that asthma affected an estimated 262 million people in 2019 and caused 461,000 deaths (1). Asthma affects both adults and children and remains as the most common chronic disease among children. In the US alone, it affects about 6 million children and young people of 0-17 years old (2).

Adolescent asthma is invariably associated with respiratory virus infection in early life since viral bronchiolitis shares many features with asthma and a subset of children develop recurrent wheezing after their initial illness (3–5). Among respiratory tract viral pathogens, respiratory syncytial virus (RSV) is the single most common viral pathogen causing respiratory tract disease in infants and children (6). Nearly all children are infected by the time of their second birthday (7, 8). Approximately 25%–40% of infants with lower respiratory tract infections (LRTIs) develop bronchiolitis or pneumonia that requires hospitalization (3, 9). Preclinical and clinical data show that infection with RSV can lead to airway remodeling and persistent airway inflammation and is closely associated with childhood recurrent wheezing and asthma (10–14). Most asthma starts with sensitization of the respiratory tract to common allergens, especially dust mites, cockroaches, animal dander, fungi, pollen, and viral infections (15). The immunohistopathologic features of asthma include epithelial injury and infiltration of inflammatory cells, consisting of eosinophils, lymphocytes, mast cells, and phagocytes (4, 16–18). It is believed that the neonatal immune system tends to favor T regulatory and Th2-type responses when microbes are first encountered. Early-life infection with respiratory viruses can disrupt normal lung development and increase the risk of chronic diseases like asthma (19). It has been shown that early infection of mice with RSV induces GATA3 expression and Th2 cytokine production (20). By promoting a Th2-type inflammatory response in the lung, RSV infection thus promotes eosinophil influx that has a critical role in allergic asthma (21–23), although a prospective cohort study of 206 previously healthy infants hospitalized with severe RSV bronchiolitis seems to suggest that Th2 phenotype plays a less-important role in subsequent immunologic development in the development of asthma or allergic sensitization (24).

Eosinophils are a subset of pleiotropic multifunctional leukocytes involved in instigation and propagation of diverse inflammatory responses, as well as modulators of innate and adaptive immunity against viral and parasitic infection (25, 26). Eosinophils are often dominant inflammatory cells present in the lungs of asthma patients, and eosinophilic inflammation is considered a characteristic feature of asthma (27). Mucus plugs in patients with asthma linked to eosinophilia and airflow obstruction (28). In mouse models, eosinophils are required for pulmonary mucus accumulation and the AHR associated with asthma (21). Eosinophil-deficient mice are significantly protected from peribronchiolar collagen deposition and increases in airway smooth muscle (22). Formalin-fixed RSV vaccine is known to induce vaccine-enhanced disease. Humans and animal models have shown that vaccination with formalin-inactivated RSV leads to prominent airway eosinophilic inflammation following RSV challenge (29). Eosinophils are usually associated with Th2-related pathologies, such as parasitic infections or allergies. IL-5, a cytokine mainly produced by Th2 and type 2 innate lymphoid cells (ILC2), stimulates eosinophils into the circulation and prolongs their survival. Chemokines such as RANTES and eotaxins are central in promoting eosinophil trafficking to the airways and airway remodeling through release of eosinophil-derived mediators such as TGF-β (4, 23). Mass spectrometry-based spectral study using formalin-fixed RSV characterized several host proteins for which expression in lung tissue is associated with an aberrant Th2-skewed response characterized by the influx of eosinophils and neutrophils (29, 30). As antigen-presenting cells (APCs), human and murine eosinophils express relevant antigen presentation membrane molecules such as CD40, CD80, and CD86 and may be induced to express major histocompatibility complex (MHC) class II (31–34). Thus, eosinophils can initiate an immune response and in turn prime T cells to increase IL-5 production by CD3/CD28 cross-linking (33, 35). The role of pulmonary eosinophilia in disease pathogenesis of allergic asthma after RSV infection is inadequately understood.

In this study, we report that eosinophils are inseparable from the induction and exacerbations of asthma after RSV infection in early life. We showed that early-life infection with RSV exacerbated allergic asthma. Adoptive transfer of asthmatic eosinophils from RSV-infected mice aggravated the pulmonary asthma pathology, which was effectively alleviated by indomethacin treatment. The expression of eosinophil surface antigen presentation-related molecules CD80, CD86, especially MHC II, was significantly upregulated when OVA antigen was encountered in adoptively transferred hosts. The study thus links eosinophils to allergic asthma in individuals with viral infection in early life.

## Materials and Methods

### Antibodies and Reagents

The following antibodies were used for flow cytometry studies: APC-labeled anti-F4/80 and FITC-labeled anti-CD11b, FITC-labeled anti-CD3 and PE-labeled anti-CD8, or FITC-labeled anti-CD3 and PE-labeled anti-CD4, PE-labeled anti-CD11C, and APC-labeled anti-Siglec-F, PerCP/Cy5.5-labeled anti-CD80, PE-Cy7-labeled anti-CD86, and PE-Cy7-labeled anti-MHC-II (eBioscience, San Diego, CA, USA; Thermo Fisher Scientific, Inc., Waltham, MA, USA) were used. To identify ILC2, lung cells were stained with PE-Cy7-labeled CD45, APC-labeled CD90.2, PE-labeled ST2, and FITC-labeled Lineage cocktail (anti-CD3, CD11b, B220, Gr-1, and TER119, eBioscience). The following antibodies were used for purification of eosinophils: APC-conjugated anti-CD19, anti-CD90.2, and anti-CD8α antibodies (BioLegend, San Diego, CA, USA) and APC-conjugated magnetic beads (MACS; Miltenyi Biotec, Auburn, CA, USA). Rabbit polyclonal antibody to major basic protein (MBP) was purchased from Affinity Biosciences (Changzhou, China) for immunohistochemical staining. OVA (A5503) and LPS (L2880, *Escherichia coli* 055:B5) were purchased from Sigma-Aldrich (St. Louis, MO, USA). Aluminum hydroxide (Inject Alum, 77161) was purchased from Thermo Fisher. Human RSV type A (A2 strain) was propagated in A549 cells as previously described (36).

### Mouse Model of Asthma

All experiments involving the use of mice were approved by the Animal Use and Care Committee of Nanjing University. Female C57BL/6 mice were maintained under standard pathogen-free conditions and used for infection and for adoptive transfer assays (approximately 150 mice in total were used for this study). To establish a model of neonatal RSV infection, mice were infected intranasally with 1.5 × 10^5^ PFU of RSV (3 µl/mouse) or with a vehicle (Dulbecco’s modified Eagles’ medium (DMEM)-containing 2% FBS) on the 7th day after birth (37).

To generate a mouse model of OVA-induced asthma, infected or uninfected mice (5-week-old, 5 per group) were intraperitoneally injected with 20 μg OVA emulsified in 0.2 ml sterile PBS containing 2 mg aluminum hydroxide on day 0 and on day 7 to induce allergen sensitization (38, 39). In LPS-induced asthma model, the mice were sensitized by intranasal instillation with 10 µl PBS containing 10 µg LPS for two times (on day 0 and day 7). The dose is within the reported endotoxin levels of atopic home samples and that of nonatopic endotoxin exposed to children (40). The mice were then challenged *via* the respiratory tract to aerosols consisting of 1% OVA or 1% LPS for 30 min each time for 9 consecutive days (from day 14 to day 22). For the challenge, mice were placed into a plastic chamber (diameter 50 cm, height 40 cm) attached to a nebulizer. Control animals were given the same volume of PBS inhalant. One day after the last allergen challenge (day 23), AHR was assessed. The animals were then euthanized, and samples were taken for analysis.

### Measurement of Airway Hyperreactivity

To assess AHR, noninvasive barometric whole body plethysmography (Emka Technologies, Paris, France) was used in conscious, unrestrained mice. The mouse was placed in the whole-body chamber to obtain a basic reading of the airway response in an average of 3 min. The airway was then stimulated with increasing concentrations of methacholine (MCh) (0, 3.1, 6.2, 12.5, 25, and 50 mg/ml) by aerosol inhalation. AHR was evaluated by enhanced pause (Penh), an indicator of bronchoconstriction (41).

### Mucus Scoring Analysis

PAS-stained lung sections were blind coded, and mucus was scored numerically (42). Scoring is as the following: 1 = Minimal/no mucus; 2 = Slight/multiple airways with goblet cell hyperplasia and mucus; 3 = Moderate/multiple airways with significant mucus and some plugging; and 4 = Severe/significant mucus plugging.

### Histopathological and Cell Counts in Bronchoalveolar Lavage Fluid

The lung tissues were separated immediately after being sacrificed on the last day of exposure. They were then fixed in 4% phosphate-buffered paraformaldehyde solution for at least 24 h and embedded in paraffin. The 4-µm histological sections were subjected to hematoxylin and eosin (H&E) staining and Periodic Acid-Schiff (PAS) staining to detect inflammatory infiltrates and mucus production. Immunohistochemical detection of eosinophils in lung tissue sections (4 µm) was performed using a polyclonal antibody to MBP. Microscopic analysis was restricted to structurally intact lung tissues in all cases under the same magnification. The cells in bronchoalveolar lavage fluid (BALF) were harvested by instillation of the lung with 1 ml ice-cold PBS and collected after centrifugation at 500×*g* for 5 min at 4°C and counted with a hemocytometer.

### Flow Cytometry Analysis

Single-cell suspensions from the lung samples were prepared by enzymatic digestion with type I collagenase (3 mg/ml) and DNase I (30 µg/ml) in RPMI-1640 medium at 37°C for 30 min. The mononuclear cells were subsequently purified by density gradient centrifugation after red blood cell lysis. The cells were incubated with corresponding antibodies at 4°C in the dark for 30 min with occasional mixing. Flow cytometry was performed with FACS Calibur with the equipped software. The data were acquired and analyzed using the FlowJo software package (TreeStar, Ashland, OR, USA).

### Quantitative PCR Analysis

Total RNA was extracted from the lung tissues using Trizol after homogenization, and the quality of RNA was assessed with the NanoDrop2000 Spectrophotometer (Thermo Fisher). The transcript levels of *IL-4*, *IL-5*, *IL-13*, and *IL17a* were determined by real-time PCR using the Applied Biosystems 7300 real-time PCR system with SYBR green PCR master mix (Q711-02-AA; Vazyme, Nanjing, China). Assays were performed in duplicate in 3 independent experiments. The mRNA levels were calculated using 2^−ΔΔCt^ method (43) and normalized to *GAPDH*. The PCR primers, listed in **Table 1**, were ordered from Sangon (Shanghai, China).

**Table 1:** List of primers used for gene expression detection (mouse).

Gene	Accession number	Forward sequence	Reverse sequence	Size (bp)
*IL-4*	NM_021283.2	5′-GGTCTCAACCCCCAGCTAGT	5′-GCCGATGATCTCTCTCAAGTGAT	102
*IL-5*	NM_010558.1	5′-CTCTGTTGACAAGCAATGAGACG	5′-TCTTCAGTATGTCTAGCCCCTG	102
*IL-13*	NM_008338.4	5′-TCCTCGCCAGACTCGTTTTC	5′-ACGGCTCCCAAGTTAGAATCT	91
*IL-17α*	NM_010552.3	5′-TTTAACTCCCTTGGCGCAAAA	5′-CTTTCCCTCCGCATTGACAC	165
*GAPDH*	NM_001289726.1	5′-ATCTCCGCCCCTTCTGCCGA	5′-CCACAGCCTTGGCAGCACCA	292

Mouse IL-4, IL-5, IL-13, and IL-17a genes were examined.

### Eosinophil Purification and Transfer

To isolate eosinophils from mice infected with RSV in early life, peripheral blood was collected from RSV-infected and allergen-induced asthmatic or mock-induced C57BL/6 mice (5 per group) after infection as induction as described. The peripheral blood was stratified into a Percoll density gradient (four densities, 1.085, 1.080, 1.075, and 1.070 g/ml) adjusted to be isotonic by adding 1 part 10 × PBS to 9 parts Percoll) following a reported protocol (33, 44). The diluted Percoll (5 ml each, in the order of high to low density) was added to 50 ml plastic tubes, and then the pooled serum diluted with PBS into 5 ml cells was added on top of the Percoll gradient. After centrifugation at 1,500×*g* for 30 min at room temperature, the eosinophils, between the 1.075 and 1.070 g/ml layers, were collected. The eosinophils were then purified by negative selection using APC conjugated anti-CD19, anti-CD90.2, and anti-CD8α antibodies and APC-conjugated magnetic beads. The purity of eosinophils (generally ≥98%) was determined by visual examination of Diff-Quick-stained cytospin preparations (44), while the viability was determined by trypan blue exclusion method.

**Figure 1:**
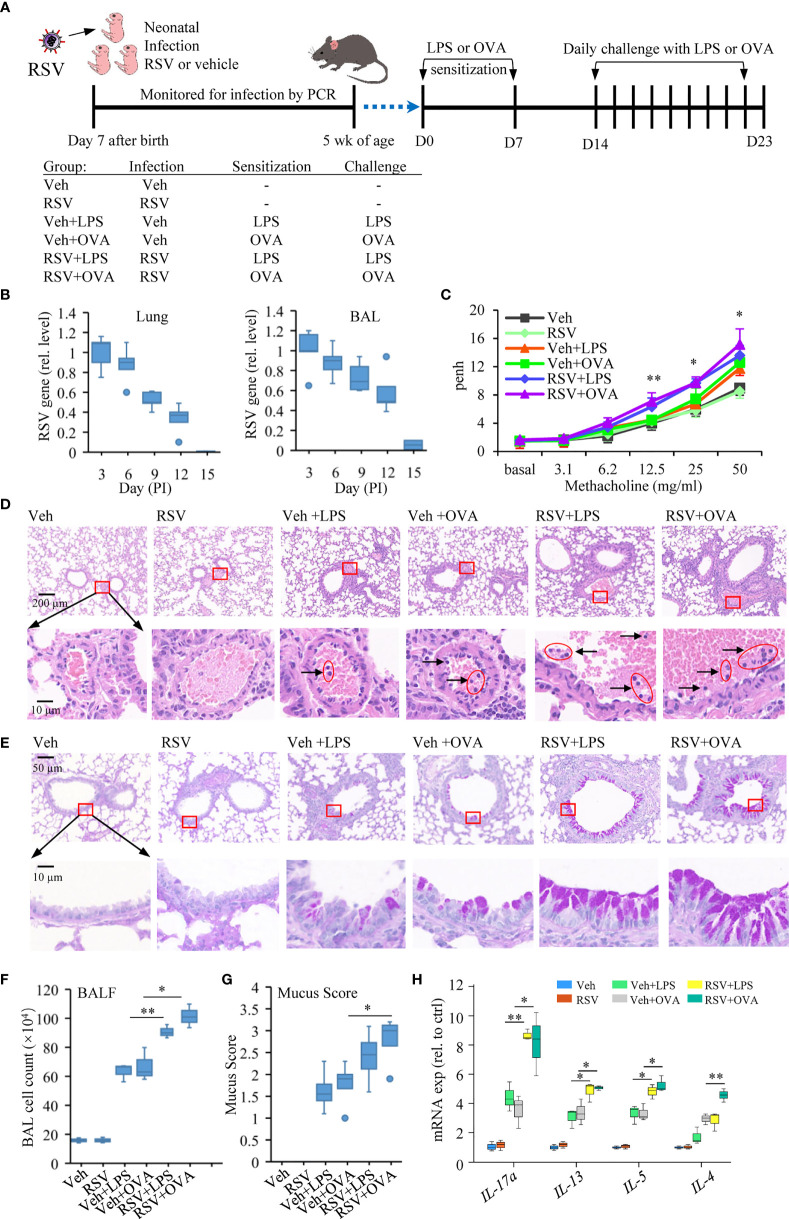
Early-life infection with RSV aggravates lung allergic pathology. **(A)** Schematic diagram and treatment regimens of experimental model of induced asthma after early-life RSV infection. Mice were infected with RSV or with a vehicle (Veh, DMEM containing 2% FBS) at 7 days of age and secondary allergen challenge initiated at 4 weeks postinfection on D0 and D7. The mice were left untreated or subjected to daily challenge with aerosolized LPS or OVA for 9 consecutive days (30 min each day). **(B)** RSV nucleocapsid gene expression was quantified in the respiratory tract (lung; BAL, cell fraction) by RT-qPCR from the day of infection until the virus is undetectable. **(C)** Airway responsiveness of mice was evaluated by Penh, which is an indicator of bronchoconstriction. The data were collected in response to gradient concentration (0, 3.1, 6.2, 12.5, 25, and 50 mg/ml) of inhaled MCh. The three sets of asterisks from left to right are RSV+OVA vs. Veh+OVA, RSV+LPS vs. Veh+LPS, and RSV+OVA vs. Veh+OVA. **(D)** Images show representative HE-stained sections from Veh-, RSV-, Veh+LPS-, Veh+OVA-, RSV+LPS-, and RSV+OVA-treated groups. The accumulation of lymphocytes in the small blood vessels around the bronchus was observed in H&E staining, which is enlarged and shown in the picture below. The scale bar is 200 μm (the upper picture) and 10 μm (the lower picture). **(E)** PAS staining was performed to detect mucus production (bright purple staining) in bronchial goblet cells. The lower picture is an enlarged view of the area inside the box in the upper picture. The scale bar is 50 μm (the upper picture) and 10 μm (the lower picture). **(F)** Total cell numbers in BALF were analyzed to evaluate the degree of airway inflammation. **(G)** Subjective mucus scoring was performed on blinded histological slides on a scale of 1–4 for mucus production (*n* = 3). **(H)** Gene expression including *IL-4*, *IL-5*, *IL-13*, and *IL-17a* in the lung tissues measured by RT-qPCR (*n* = 3). Statistical results are shown: ^*^p < 0.05 and ^**^p < 0.01.

After the test animals were lightly anesthetized with 1% isoflurane, purified eosinophils (1 × 10^7^/20 µl) or vehicle control (normal saline, 20 µl) were adoptively transferred to the lungs of recipient mice by means of intranasal instillation administration (45). The transfer of eosinophils (or saline vehicle) was completed within 1 h after intraperitoneal injection of OVA. The timeline of these experimental operations is shown in **Figure 1A**, including OVA sensitization/challenge and adoptive transfer schedule. Indomethacin (30 mg/kg, i.p., *n* = 5) was administered 60 min before antigen challenge to inhibit eosinophil accumulation to airway in the therapy group (46).

### Statistical Analysis

Graphical representation and statistical analyses were performed using Excel software. For two-group comparisons, a two-way ANOVA was performed, followed by Tukey’s *post-hoc* test to determine significant differences between groups (ns, no significance; ^*^p < 0.05, ^**^p < 0.01, and ^***^p < 0.001).

## Results

### Early-Life Infection With RSV Leads to Exacerbated Pulmonary Allergic Pathology

To evaluate the effect of prior RSV infection on allergic response later in life, neonatal mice were infected intranasally with RSV (1.5 × 10^5^ PFU) or with a vehicle on the 7th day after birth. The mice were then sensitized with LPS or with OVA, allergens that induce allergic asthma in mice (47, 48), *via* intraperitoneal injection twice at 5 weeks of age (D0) and 1 week afterward (D7) (**Figure 1A**). To elicit an allergic response, mice were challenged with aerosolized LPS or OVA for 9 consecutive days of 30 min exposure each time. RSV in the lungs after the infection was monitored by detection of RSV nucleocapsid gene expression in the lung tissues and in the BAL. On day 15, RSV was cleared from the infected mice (**Figure 1B**). One day after the last challenge (D23), mice were subjected to the measurement of AHR using whole body plethysmography. Mice sensitized with LPS or OVA had moderate AHR (Veh+LPS or Veh+OVA group). Early-life infection with RSV exacerbated asthmatic response to LPS or OVA stimulation (RSV+LPS or RSV+OVA group) (**Figure 1C**). Histological staining of lung tissue sections showed that RSV+LPS or RSV+OVA mice had more severe pathological damages. More lymphocytes were observed in the small blood vessels around the bronchus in these samples (**Figure 1D**). Hyperplasia of goblet cells with mucus overproduction is a feature of asthma (49). PAS staining showed that more severe bronchial wall thickening and mucus production of epithelial goblet cells in their lungs of asthmatic mice with prior RSV infection compared with those given only LPS or OVA (**Figure 1E**). Similarly, BALF had more cell infiltration (**Figure 1F**) and higher mucus scores in the PAS-stained lung sections of RSV+OVA or RSV+LPS group (**Figure 1G**).

In addition, the expression of *IL-4*, *IL-5*, *IL-13*, and *IL-17a*, a Th17 cytokine that synergizes with IL-13 to activate Th2 cells (50), was significantly increased in the lungs of RSV+OVA or RSV+LPS compared with those in the Veh+LPS or Veh+OVA group (**Figure 1H**). These results indicated that early-life infection of mice with RSV was a contributing factor for induced asthmatic disease.

### Early-Life Infection With RSV Predisposes Eosinophil Pulmonary Accumulation

**Figure 2:**
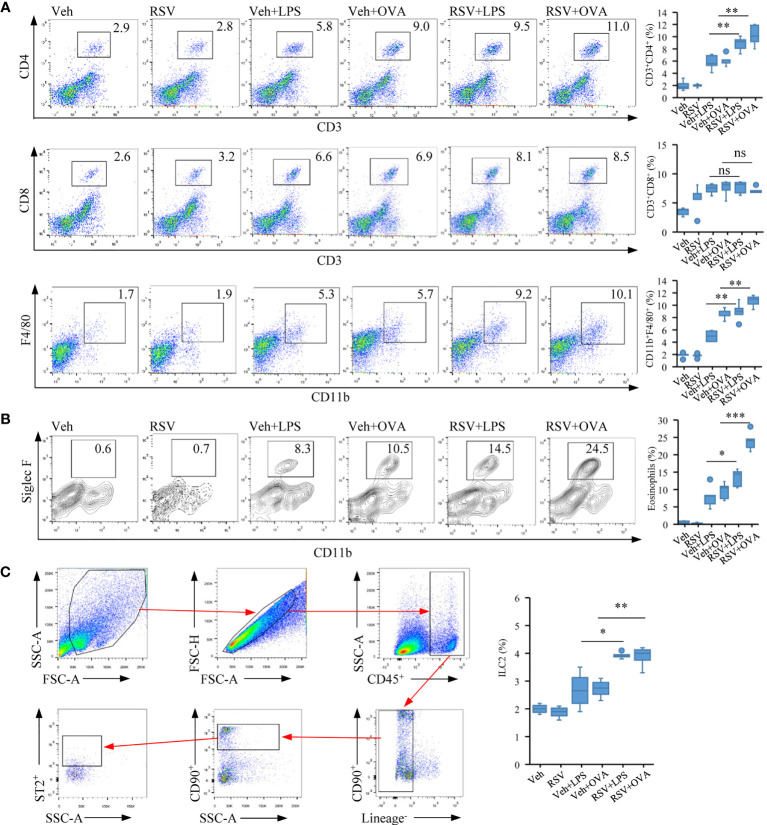
Early-life RSV infection predisposes eosinophil accumulation and allergic immune response. **(A)** Representative staining of CD4^+^ T (CD3^+^CD4^+^), CD8^+^ T (CD3^+^CD8^+^) and macrophage (F4/80^+^CD11b^+^) in the lung tissues by flow cytometry analysis (*n* = 5). **(B)** Representative staining of eosinophil cells (CD11c^−^CD11b^+^SiglecF^+^) in the lung tissues by flow cytometry analysis (*n* = 5). **(C)** Gating strategy for ILC2 cells (CD45^+^CD90.2^+^ST2^+^Lineage^−^). Quantification of CD4^+^ T, CD8^+^ T, macrophage, eosinophil, and ILC2 cell populations are presented (*n* = 5). The statistical box plots are shown: ns, no significance; ^*^p < 0.05, ^**^p < 0.01, and ^***^p < 0.001.

We examined immune cell populations in the lungs. Marked increases in both CD4^+^ T and macrophages, but not CD8^+^ T cells, were detected in the RSV+LPS and the RSV+OVA mice compared with that in the Veh+LPS and the Veh+OVA mice (**Figure 2A**). The number of CD11c^−^CD11b^+^Siglec F^+^-positive eosinophils was also increased significantly in the lungs of RSV-infected LPS/OVA-induced asthmatic mice compared with that only receiving LPS or OVA (*p* < 0.001) (**Figure 2B**). ILC2 belongs to an expanding family of innate lymphocytes that is a potent source of immune effector cytokines at the initiation of an immune response (50). Accordingly, the percentages of ILC2 (the gating strategy for CD45^+^CD90.2^+^ST2^+^Lineage^-^ is provided in **Figure 2B**) were increased in RSV+LPS and in RSV+OVA mice compared with that of the LPS and OVA groups (**Figure 2C**).

The results thus showed that neonatal infection with RSV exacerbated lung pathology in mice and pulmonary eosinophil accumulation after allergic stimulation later in life.

### Adoptive Transfer of Asthmatic Eosinophils Leads to Pulmonary Damage

**Figure 3:**
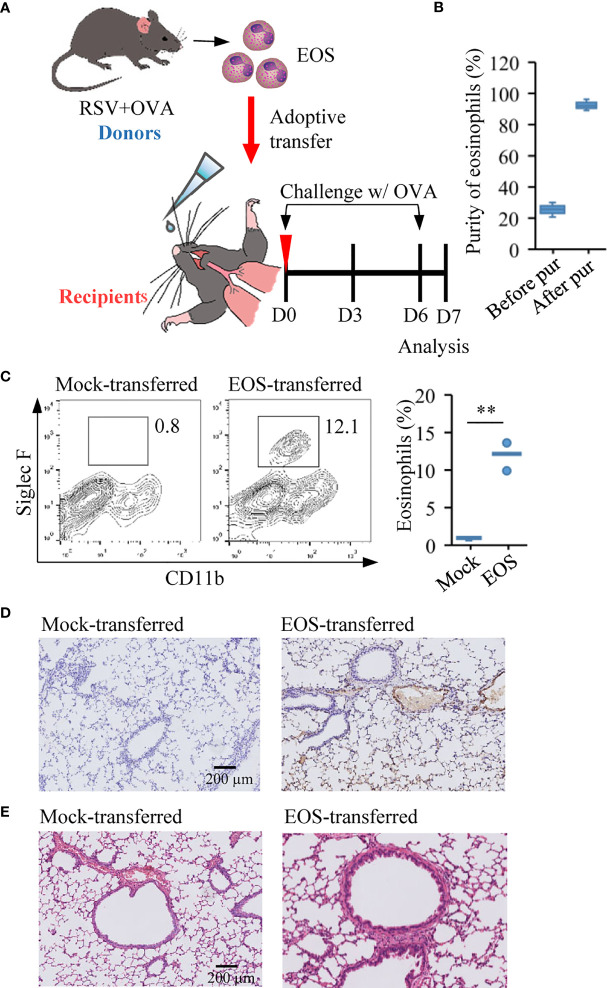
Adoptive transfer of asthmatic eosinophils leads to pulmonary damage. **(A)** Experimental design of adoptive transfer of eosinophils from RSV+OVA mice to recipient mice. The mock-transferred mice were instilled into the nasal with vehicle control (normal saline/20 µl) and the EOS-transferred mice were instilled with highly purified eosinophils (1 × 10^7^ cells/20 µl) from RSV+OVA mice. After adoptive transfer, the two groups of mice were directly challenged with OVA aerosol inhalation to give a foreign antigen stimulation. **(B)** The purity of eosinophils was evaluated by visual examination of Diff-Quick-stained cytospin preparations. **(C)** The presence of eosinophils after adoptive transfer was validated by flow cytometry. Quantification of eosinophil are presented as box plots (*n* = 5), ^**^p < 0.01. **(D)** Immunohistochemical detection of eosinophils in lung tissue sections was performed by detected MBP protein. The scale bar is 200 μm. **(E)** Images show representative H&E-stained sections from lung of mock-transferred and EOS-transferred mice. The scale bar is 200 μm.

Eosinophils are crucial circulating granulocytes in the pathogenesis of allergic diseases like asthma (25). They participate in the modulation of immune response, induction of airway hyperresponsiveness, and remodeling, characteristic features of asthma (51, 52). Due to significant accumulation of eosinophils in the asthmatic mice with prior RSV infection, we would like to explore the specific contribution of eosinophils to lung airway inflammatory process. Eosinophils from RSV+OVA asthmatic mice were separated from the peripheral blood, and highly purified eosinophils were adoptively transferred to naive mice (1 × 10^7^ cells/mouse in 20 µl) by direct instillation into the tracheas of recipient mice (**Figure 3A**). Mice received normal saline instillation were defined as mock-transferred control. The purity of eosinophils, determined by Diff-Quick staining method, was normally ≥98% (**Figure 3B**). Within 1 h after adoptive transfer, mice in these two groups were directly challenged with OVA *via* aerosol inhalation to give a foreign antigen stimulation (**Figure 3A**). As shown in **Figure 3C**, the number of eosinophils in the lung tissues of EOS-transferred mice was increased significantly compared with mock-transferred mice (**Figure 3C**). We also performed immunostaining study to check the release of MBP, an eosinophilic protein responsible for tissue damage, exfoliation, and bronchospasm in allergic diseases such as asthma (53). Data showed the presence of MBP protein produced by eosinophils in the interstitium of the alveoli in the transferred mice, but not in mock-transferred mice (**Figure 3D**). Histopathological assessment showed slight inflammatory infiltration in adoptively transferred mice compared with that in mock-transferred mice (**Figure 3E**). These results strongly indicated that eosinophils from asthmatic mice possessed tissue damage and proinflammatory effect.

### Adoptive Transfer of Asthmatic Eosinophils Results in Increased Susceptibility to Allergen Stimulation

**Figure 4:**
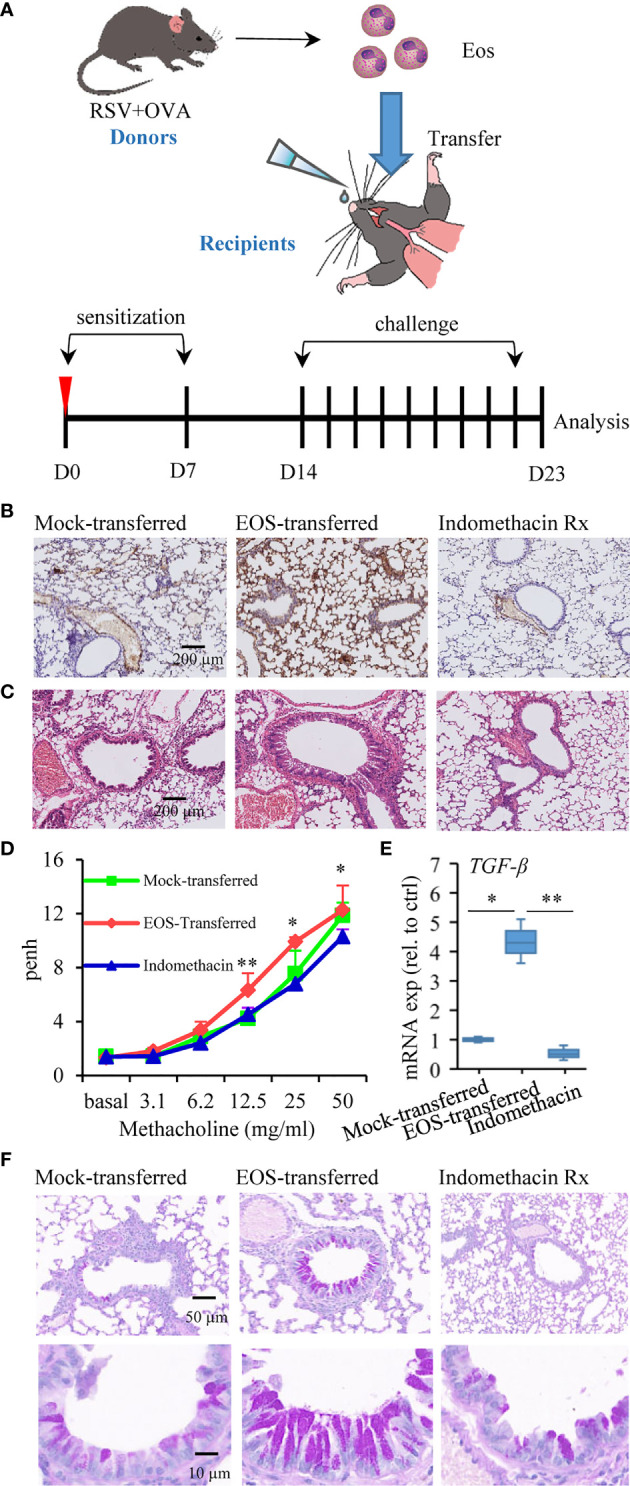
Adoptive transfer of asthmatic eosinophils results in increased susceptibility to allergen stimulation. **(A)** Schematic timeline of the experimental model of asthma mice after being transferred, in which mice were treated by OVA sensitization and challenge. **(B)** MBP protein immunohistochemistry staining was used to detect the presence of eosinophils in lung tissue sections of mock-transferred, EOS-transferred, and indomethacin-treated mice. The scale bar is 200 μm. **(C)** Images show representative H&E-stained sections from each group. The scale bar is 200 μm. **(D)** AHR of mice is measured by monitoring Penh in response to gradient concentration (0, 3.1, 6.2, 12.5, 25, and 50 mg/ml) of inhaled MCh. The three sets of asterisks from left to right are EOS-transferred vs. mock-transferred, EOS-transferred vs. mock-transferred, indomethacin vs. EOS-transferred. **(E)** *TGF-β* expression in the lung tissues measured by RT-qPCR (*n* = 3). **(F)** PAS staining was performed to monitor mucus production in bronchial goblet cells (bright purple staining). The scale bar is 50 μm (the upper picture) and 10 μm (the lower picture). *p < 0.05, and **p < 0.01.

In view of the role of eosinophils in asthma and signs of lung damage in RSV+OVA-challenged mice from this study, we would like to further explore the specific contribution of eosinophils to asthma. We thus investigated whether transferred eosinophils from RSV-induced asthmatic mice possessed proasthmatic activity after allergen stimulation. In this regard, mice were injected with OVA on day 0 and day 7 after adoptive transfer of eosinophils from asthmatic mice (**Figure 4A**). One week later, the mice were challenged with OVA aerosols for 9 consecutive days to elicit an allergic asthmatic response (**Figure 4A**). Indomethacin (30 mg/kg, ip) as a control was administered 60 min prior to antigen challenge since indomethacin was known to inhibit eosinophil accumulation to the airway (46). A significant eosinophil infiltration by MBP protein staining was detected in the lung tissues of EOS-transferred mice compared with mock-transferred asthmatic mice, and indomethacin treatment effectively inhibited eosinophil accumulation (**Figure 4B**). Similar to that observed in RSV-infected and allergen-sensitized and allergen-challenged mice, mice in adoptively transferred group had severe pathology and bronchial wall thickening in the lung tissues (**Figure 4C**). Examination of the pulmonary ventilation function of asthmatic mice showed that EOS-transferred asthmatic mice had enhanced airway resistance (**Figure 4D**). TGF-β-induced epithelial mesenchymal transition (EMT) leads to disruption of mucosal barrier function (54). Consistently, higher levels of TGF-β were detected in EOS-transferred asthmatic mice (**Figure 4E**). In PAS-stained sections, severe mucus production was detected in adoptively transferred mice compared to that in mock-transferred asthmatic mice (**Figure 4F**). Indomethacin treatment ameliorated the symptoms including airway resistance, tracheal wall thickening and mucus secretion and in EOS-transferred mice (**Figures 4C, D, F**). These results demonstrated that eosinophils from asthmatic mice with a history of RSV infection were responsible for pathological damage associated with allergic respiratory inflammation.

### Lung Eosinophil Has Increased Expression of MHC II, CD80, and CD86 After Adoptive Transfer

**Figure 5:**
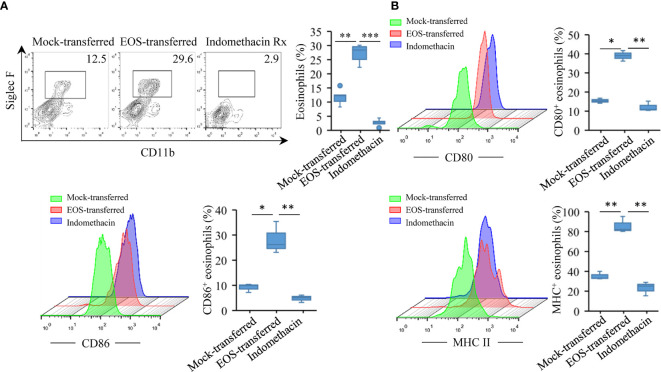
Increased expression of MHC II, CD80, and CD86 molecules on lung eosinophils after adoptive transfer. **(A)** Using flow cytometry to verify the number of eosinophils in the lungs of three groups of mice. Quantification of eosinophils are presented as box plots (*n* = 5). **(B)** CD80, CD86, and MHC II expression on eosinophil surface were monitored by flow cytometry in lung tissues of mice. Quantification of molecules are presented as box plots (*n* = 3). Statistical results are shown: ^*^p < 0.05, ^**^p < 0.01, and ^***^p < 0.001.

In asthmatic patients and experimental models, eosinophils have been demonstrated to express MHC II and costimulatory molecules necessary to act as APCs (33, 55, 56). We next explored whether murine eosinophils from the airways of antigen-sensitized and antigen-challenged mice with a history of RSV infection expressed higher level of molecules involved in the presentation of exogenous antigens. To this end, we detected immunostimulatory ligand antigen presentation-related molecules such as CD80 and CD86, two B7 proteins with recognized roles as costimulatory signals for T-cell responses (57), from the lung tissues of the animals. Flow cytometry study showed that eosinophil population from the lung tissues increased significantly after adoptive transfer (**Figure 5A**). For comparison, mice that received OVA aerosol inhalation only had low levels of CD80 and CD86 expression on eosinophils, while MHC II expression was undetected. In EOS-transferred mice, CD80, CD86, and MHC II expression had significant increases on the eosinophils (**Figure 5B**). Indomethacin treatment effectively reduced the expression of the molecules (**Figure 5B**). These data suggested that eosinophils from RSV-infected mice could function as APC responsible for induced asthma.

### Adoptive Transfer of Asthmatic Eosinophils Enhances Proinflammatory Response

**Figure 6:**
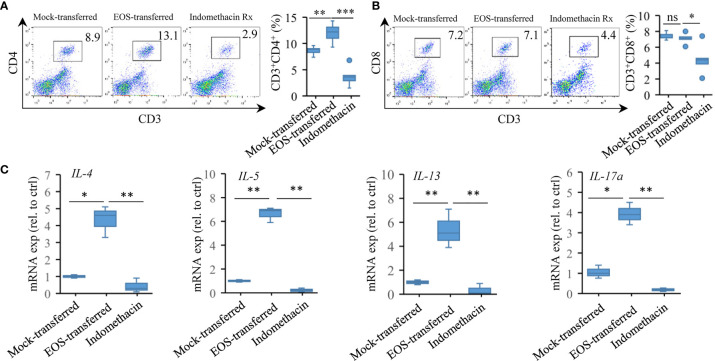
Adoptive transfer of asthmatic eosinophils enhances proinflammatory response. **(A)** CD4^+^ and **(B)** CD8^+^ T-cell accumulation was detected by FACS (*n* = 5). **(C)** Gene expression of *IL-4*, *IL-5*, *IL-13*, and *IL-17a* in the lung tissues of mock-transferred, EOS-transferred, and indomethacin-treated mice were measured by RT-qPCR (*n* = 3). Statistical results are shown: ns, not significant; ^*^p < 0.05, ^**^p < 0.01.

The effector T cells promote immune responses related to the diseases, such as the production of airway Th2 cytokines and the induction of lung pathology (58, 59). We then checked the recruitment of T cells after adoptive transfer of eosinophils. In mock-transferred mice that did not receive intranasal eosinophil instillations, only low percentages of CD3^+^CD4^+^ and CD3^+^CD8^+^ cells were present in the lung tissues detected by flow cytometry assessment. Adoptively transferred mice that received RSV-exposed eosinophils had a significant increase in CD3^+^CD4^+^ cell population in lung tissues (**Figures 6A, B**). Low level of CD3^+^CD8^+^ cells were detected in those mice. Indomethacin treatment resulted in reduction in both CD3^+^CD4^+^ and CD3^+^CD8^+^ T-cell populations (**Figure 6A**). Compared with mock-transferred mice, the expression of the Th2 cytokines like *IL-4*, *IL-5*, *IL-13*, and Th17 cytokine *IL-17a* was significantly increased in the lungs of mice receiving asthmatic eosinophils (**Figure 6C**). Consistently, the expression of these cytokines was markedly reduced in indomethacin-treated samples (**Figure 6C**), indicating a severe Th2 response in asthmatic pathology of transferred mice.

Together, the results demonstrated that pulmonary eosinophils were responsible for recruitment of effector T cells and for enhanced proinflammatory response in aggravated allergic asthma associated with early-life infection with RSV.

## Discussion

More than 80% of children experience at least one RSV infection by the age of 2, of which the majority occurs in the first year of life (60). While the majority of infants present only mild upper respiratory tract infection (URTI) or occasionally otitis media, around 1/3 will develop an infection of the LRTI, usually bronchiolitis. Airway inflammation is central to disease pathophysiology and relates to airway dysfunction caused by airway remodeling and stimulation of inflammatory mediators (54). TGF-β-induced EMT leads to airway remodeling, wall thickening, and disruption of mucosal barrier function as a consequence of severe RSV LRTIs (14). Airway abnormalities and preexisting airway damage due to RSV may increase susceptibility to allergens, increasing the risk of asthma later in life (61). Here, we show that the eosinophils correlate with the induction and exacerbation of asthma after RSV infection in early life. Mice infected with RSV early in life had significantly severe asthma symptoms, including rich mucus production in the bronchi, increased eosinophil numbers, higher AHR, and allergic airway lymphocyte infiltration. Eosinophil adoptive transfer of asthma mice induced by RSV infection in early life aggravated the pulmonary asthma pathology. In addition, the expression of eosinophil surface antigen presentation-related molecules was significantly upregulated when OVA antigen was encountered again. It also leads to a significant increase in the accumulation of CD4^+^ T cells in the lungs. Treatment of adoptively transferred asthma mice with indomethacin, an inhibitor of inflammation and eosinophil infiltration (46), alleviated pulmonary allergic pathology.

Pulmonary eosinophils are believed to play a crucial role in the pathogenesis of asthma and other allergic diseases (62, 63). Lung eosinophilia also causes proinflammatory response during SARS-CoV2 infection and is a contributing factor to vasculitis in COVID-19 disease (64, 65). We detected elevated expression of a number of cytokines regulate the function of eosinophils and other cells in asthma. During allergic inflammation, IL-5, IL-3, and GM-CSF could promote eosinophil survival, thereby facilitating accumulation of eosinophils in the airways (66). Cytokines, growth factors, and cytotoxic granule proteins, such as CCL11, TGF-β, MBP, and ECP produced or released by activated eosinophils could lead to tissue damage and airway remodeling (67, 68). CCR3 is a relatively selective chemokine receptor for eosinophils, together with its ligand eotaxins, such as CCL11, CCL24, and CCL26 induce eosinophil migration from the blood to the diseased tissue. Thus, this study links Th2 cytokine production to eosinophil function in allergic asthma associated with early-life infection by respiratory viruses.

Activated eosinophils have an antiviral role in the immune response. Eosinophils express several toll-like receptors (TLRs) and retinoic acid-inducible gene I (RIG-I)-like receptor (69, 70), which are able to recognize pathogen-associated molecular patterns (PAMPs). Some of these receptors, such as TLR3, a double-stranded RNA receptor, are reduced in eosinophils of allergic rhinitis patients, provide a link between viral infection and allergic exacerbation (71, 72). Eosinophils have been found to accumulate and become activated in airways of asthma patients or murine models during virus-induced exacerbations, manifested by increased expression of CD80, CD86, and MHC II (26, 73). Interestingly, the release of eosinophil cationic protein was not induced by RSV infection, but the capacity of eosinophils to capture virus was reduced with increasing severity of asthma (74).

Eosinophils in the airway lumen are capable of traversing the respiratory epithelium, entering the tissues around the trachea, and being transferred to the lymph nodes (33). The instillation of peripheral blood eosinophils of RSV-infected asthmatic mice into the lungs of naïve mice verified this fact that the infiltration of eosinophils in the lung tissues was significantly increased after adoptively transferred. Adoptive transfer leads to enhanced asthma pathological response in mice, including increased bronchial mucus secretion and tube wall thickening, indicating that eosinophils are directly related to the enhanced asthmatic inflammatory response in RSV infected asthma mice model.

**Figure 7:**
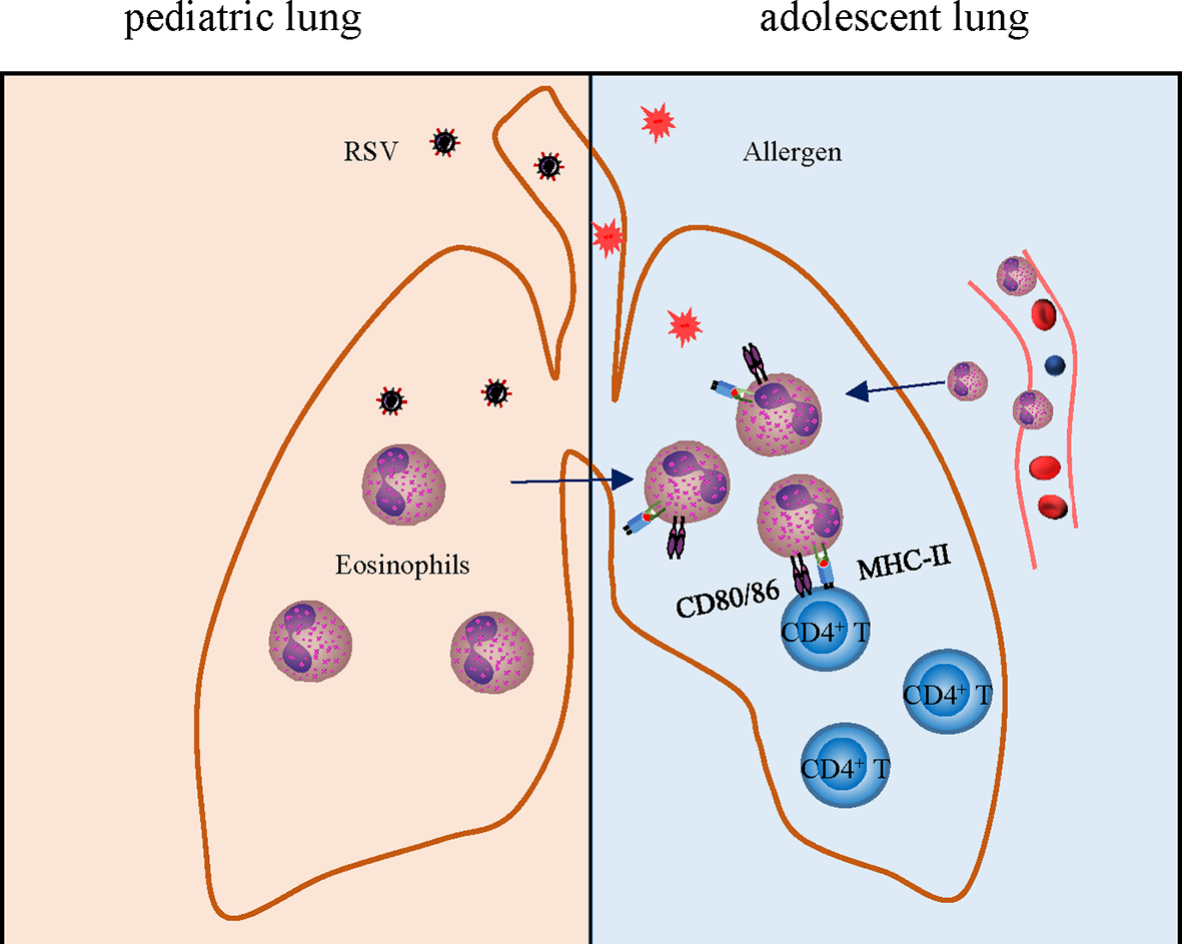
Schematic drawing showing the role of eosinophils in enhanced allergic asthma after early-life infection with RSV. Early-life infection with RSV leads to an increase of eosinophils leading to asthma pathology aggravation later in life, which links to airway remodeling, enhanced antigen presentation, and inflammation.

In addition, the capacity of eosinophils to serve as APCs has received extensive attention in asthma. A critical test was to determine whether eosinophils within the airways could function to present antigens and elicit T-cell responses *in vivo* after encountering inhaled antigens. We found that the expression of antigen presentation related molecules CD80, CD86 especially MHC II, on the surface of eosinophils was markedly upregulated when encountered OVA antigen again (**Figure 7**). Accumulation of CD4^+^ T cells after adoptive transfer also provided evidence that the enhanced antigen presenting function of eosinophils leads to aggravated inflammatory response.

Our data support a conclusion that the increased eosinophils can act as APC since adoptively transferred mice that received RSV-exposed eosinophils had a significant increase in CD3^+^CD4^+^ cell population in the lung tissues. It is also possible that transferred eosinophils could in some way be just enhancing the ability of other APCs in the recipients to function or alter the permeability of the mucosa to antigens. Further studies will be needed using mice congenitally deficient in eosinophils. We showed both OVA and LPS induced asthmatic response in mice pre-exposed to RSV in early life. RSV infection may also be a predisposing factor to induced asthma to air pollutants and particulate matters.

Human eosinophils express Toll-like-receptors for RNA and for LPS signaling (69), thus could have a direct effect on induced allergic reaction. We detected increased expression of APC-related molecules in eosinophils from EOS-mice. It is interesting to know if trained immunity or epigenetic factors play a role in eosinophil memory and their response. Nonetheless, our study shows that eosinophils play an indispensable role in the occurrence and development of allergic asthma after early-life infection with respiratory viruses.

## Data Availability Statement

The raw data supporting the conclusions of this article will be made available by the authors, without undue reservation.

## Ethics Statement

The animal study was reviewed and approved by Animal Use and Care Committee of Nanjing University.

## Author Contributions

EL and DZ conceived the ideas. DZ, JY, YZ, LW, and SH designed and performed the experiments. JS and GY provided reagents. DZ, GY, JY, SH, and EL analyzed the data. DZ and EL curated the data and wrote the manuscript. DZ, SH and EL edited the manuscript. All authors listed have made a substantial, direct, and intellectual contribution to the work and approved it for publication.

## Funding

The work was supported by grants from the NSFC (81871636 to EL), from Jiangsu Natural Science Foundation (BK20200316 to SH), from Central Universities Fundamental Research Funds (14380470 to SH), and from Science, Technology and Innovation Commission of Shenzhen Municipality (JSGG20200519160755008 to EL).

## Conflict of Interest

The authors declare that the research was conducted in the absence of any commercial or financial relationships that could be construed as a potential conflict of interest.

## Publisher’s Note

All claims expressed in this article are solely those of the authors and do not necessarily represent those of their affiliated organizations, or those of the publisher, the editors and the reviewers. Any product that may be evaluated in this article, or claim that may be made by its manufacturer, is not guaranteed or endorsed by the publisher.
